# Combined analysis of plasma metabolome and intestinal microbiome sequencing to explore jiashen prescription and its potential role in changing intestine–heart axis and effect on chronic heart failure

**DOI:** 10.3389/fcvm.2023.1147438

**Published:** 2023-03-10

**Authors:** Xialian Cui, Yangyan Su, Xiaotong Huang, Jiaping Chen, Jiang Ma, Peiran Liao, Xin He

**Affiliations:** ^1^School of Traditional Chinese Medicine, Guangdong Pharmaceutical University, Guangzhou, China; ^2^Guangdong Provincial Key Laboratory of Advanced Drug Delivery, Guangdong Provincial Engineering Center of Topical Precise Drug Delivery System, Guangdong Pharmaceutical University, Guangzhou, China

**Keywords:** heart failure, jiashen prescription, intestinal microbiota, plasma metabolomics, weighted gene co-expression network analysis (WGCNA)

## Abstract

**Background:**

Heart failure (HF) is a syndrome with global clinical and socioeconomic burden worldwide owing to its poor prognosis. Jiashen Prescription (JSP), a traditional Chinese medicine (TCM) formula, exhibits unambiguous effects on treating HF. Previously, we have reported that underlying mechanisms of JSP by an untargeted metabolomics approach, but the contribution of gut microbiota and metabolic interaction to the cardioprotective efficacy of JSP remains to be elucidated.

**Materials and methods:**

Firstly, the rat model of heart failure was established by the permanent ligation of the left anterior descending coronary artery. The efficacy evaluation of JSP in treating HF rats was per-formed by left ventricular ejection fraction (LVEF). Then, 16S rRNA gene sequencing and LC/MS-based metabolomic analysis were utilized to explore the characteristics of cecal-contents microecology and plasma metabolic profile, respectively. After that, the correlation between intestinal micro-ecological characteristics and plasma metabolic characteristics was analyzed to explore the potential mechanism of the JSP treatment in HF.

**Results:**

JSP could improve the cardiac function of heart failure rats and thus ameliorate heart failure *via* enhancing rat LVEF. Results of intestinal flora analysis revealed that JSP not only adjusted gut microbiota disturbances by enriching species diversity, reducing the abundance of pathogenic bacteria (such as *Allobaculum, Brevinema*), as well as increasing the abundance of beneficial bacteria (such as *Lactobacillus, Lachnospiraceae_NK4A136_group*), but also improved metabolic disorders by reversing metabolite plasma levels to normality. Through the conjoint analysis of 8 metabolites and the OTUs relative abundance data in the 16srRNA sequencing results by WGCNA method, 215 floras significantly related to the eight compounds were identified. The results of the correlation analysis demonstrated a significant association between intestinal microbiota and plasma metabolic profile, especially the significant correlation of *Ruminococcaceae_UCG-014* and Protoporphyrin IX, *Ruminococcaceae_UCG-005, Christensenellaceae_R-7_group* and nicotinamide, dihydrofolic acid.

**Conclusion:**

The present study illustrated the underlying mechanism of JSP to treat heart failure by affecting intestinal flora and plasma metabolites, provide a potential therapeutic strategy against heart failure.

## Introduction

Heart failure (HF) is a chronic progressive disease (1), which is a complex group of clinical signs of impaired ventricular filling or ejection capacity due to any structural or functional abnormality of the heart. It is the end-stage manifestation of various cardiovascular diseases (CVDs), which bring high prevalence and mortality and threaten human health (2). Thus, discovering novel mechanisms of HF and identifying potential therapeutic targets are extremely important ways of preventing heart failure. Recently, several studies indicated that the intestine microbiota can influence the cell and organ functions of the host and multiple mechanisms and pathways of diseases (3). Among them, several studies have shown that intestinal microbes may affect the cardiovascular system, and the concept of the “intestine–heart axis” has been gradually applied to explore the treatment of CVD and heart failure (4, 5).

The intestinal microbiome transmits information to the distant organs of the host through various biochemical signals and metabolites. In actuality, the intestinal microbiome can affect the cardiovascular system. First, some studies found that the flora of intestine microbiota in patients with heart failure decreased significantly (6). This may lead to an imbalance between beneficial and harmful microorganisms. The former can produce many beneficial metabolites, such as short-chain fatty acids, while the latter may bring a large number of harmful metabolites, such as primary bile acid and trimethylamine oxide (TMAO) (7). These metabolites can directly or indirectly affect the heart after entering the circulatory system. Then, heart failure can lead to structural and functional abnormalities of the heart, which result in decreased cardiac output and tissue perfusion. In turn, it allows bacteria and lipopolysaccharide to translocate into systemic circulation, which induces the inflammation and accelerates the development of heart failure (8). Therefore, through the study of intestinal microbiota, it is helpful to study the therapeutic mechanism of drugs for heart failure or cardiovascular disease from the perspective of the intestine–heart axis. It was proposed that treatment with *Lactobacillus rhamnosus* GR-1 as a probiotic could delay the development of heart failure after coronary artery occlusion in rats (9). Methanogens can treat CVD by reducing plasma levels of TMAO (10). Phenaceglutamide, a metabolite of intestinal microbiome negatively correlated with pulse wave velocity and systolic blood pressure, could also be a potential therapeutic target (11).

Jiashen prescription (JSP) is a clinical prescription used for treating heart failure, which is established under the guidance of the theoretical thought of traditional Chinese medicine (TCM). It is mainly prepared from several Chinese medicinal materials, including *Astragali Radix, Salviae Miltiorrhizae Radix et Rhizoma, Periplocae Cortex, Notoginseng Radix et Rhizoma, Leonuri Herba, Citri Reticulatae Pericarpium, Cinnamomi Ramulus*, and *Descurainiae Semen Lepidii Semen* (12). Previous chemical studies showed that there were at least 68 chemical compounds identified from JSP, mainly including phenolic acids, tanshinones, flavonoids and their glycosides, cardiac glycosides, triterpene saponins, and C21 steroids (13). From the perspective of modern pharmacological effects, this prescription has the effects of improving heart hemodynamics, enhancing heart function, inhibiting activation of renin–angiotensin, diuresis, and inhibiting ventricular remodeling in animals with heart failure (14). However, TCM has the characteristics of multi-components and multi-targets. When taking Chinese medicine orally, the most of active ingredients in TCM cannot directly enter the blood system to exert their effects but may be directly or indirectly metabolized by the intestinal microbiome and then enter the blood system to achieve the therapeutic effect on diseases (15).

Thus, intestinal microflora has become a new and important frontier in the understanding of TCM (16). At present, studies have shown the influence of intestinal microbiota on the metabolism of bioactive ingredients of TCM (17). Not only polysaccharides (18) but also some macromolecular saponins, terpenes, and alkaloids have been effectively transformed by intestinal microbiome. Then, bioavailability and therapeutic activity are increased as well (19). In the treatment of chronic syndrome and glycolipid metabolic diseases, which still are global health problems, TCM has its unique advantages (20, 21). One of the main therapeutic mechanisms is to increase the relative abundance of beneficial bacteria by improving the intestinal environment (22).

Evidently, in this study, the rat model of heart failure was used to investigate the treatment effect of JSP, which was established by the ligation of the left anterior descending coronary artery (LAD) (23). In the previous study of our research group, Miao et al. found that JSP improved the cardiac function of heart failure rats and thus ameliorated heart failure *via* enhancing rat LVEF and LVFS and decreasing LVIDd, LVIDs, IVSd, and IVSs. Based on the biochemical analysis and histopathological examination, it was found that JSP could reduce the markers levels of heart failure and myocardial damage that included serum lactate dehydrogenase (LDH) activity and the level of NT-pro BNP and inhibit myocardial fibrosis (24). In this study, we collected and sequenced the intestinal contents of rats in each treatment group in the previous study. For this purpose, the blood metabolites of rats in different treatment groups were analyzed and screened by the fuzzy C-means clustering method. Then weighted correlation network analysis was used to jointly analyze these metabolites and 16S rRNA high-throughput sequencing data of intestinal microbiome in each treatment group, to find metabolites that may be related to the JSP treatment effect and their significantly related intestinal microorganisms, to clarify the mechanism of JSP treatment through the “intestine–heart axis”.

## Materials and methods

### Experimental reagents

Jiashen prescription was produced by Tasly Pharmaceutical Group Co., Ltd., Tianjin, China. Captopril (CPT) was purchased from Shanghai Pharmaceutical Group Corp., (Shanghai, China). DNA extraction kits were purchased from Macherey-Nagel (Düren, German), and HPLC-grade methanol and acetonitrile were obtained from Merck (Darmstadt, Germany). Formic acid and 2-chlorophenylalanine were bought from Thermo Fisher Scientific (MA, USA).

### Animals and experimental protocols

All experiments were in accordance with the guidelines for laboratory animal care and use, and the procedures were approved by the Research Ethical Committee of Guangdong Pharmaceutical University (Guangzhou, China). A total of 46 SPF (SD) rats (license NO. SCXK (yue) 2018–0002), weighing from 180 to 220 g, were purchased from the Guangdong Medical Laboratory Animal Center (Guangzhou, China). The animals were housed at 20–25°C, 40–70% humidity and 12 h dark/light cycle conditions with free access to a standard chow diet, and tap water *ad libitum*. All rats were exposed to an ‘adaptive feeding' paradigm for a week before the start of experiments.

The experiment included four groups (*n* = 6 per group), namely Control group, Model group, JSP group, and CPT (Captopril) group. In the control group (*n* = 6), the rats were subjected to the same surgeries except for the ligation. Heart failure symptoms were induced by carrying out ligation of the left anterior descending coronary artery of the rats as previously reported (25), and the chronic congestive heart failure model was established after 4 weeks of operation and normal feeding. The left ventricular ejection fraction (LVEF) of HF rats was < 60%, and it has been considered that the chronic HF rat models were successfully established (26). JSP and CPT were orally administered to the rats at 3 and 0.05 g/kg/day, which was selected based on their human equivalent dose used in clinical practice. In the previous research in our laboratory, this dose of JSP had been used to prove the efficacy of JSP (24).

In total, 40 rats underwent LAD ligation to establish a heart failure model. Among the 40 rats, eight rats died during the operation and the other eight rats did not meet the criteria of chronic heart failure after the operation; the success rate of the heart failure model rats was 60%. Therefore, 18 rats (*n* = 18) with similar body weight were randomly divided into three groups as follows: (1) HF group, the rats received LAD ligation to induce HF, (2) JSP group, a dose of 3 g/kg/day JSP was given the corresponding drugs by gavage for 4 weeks, and (3) CPT group, a dose of 0.05 g/kg/day captopril was gavage to rats for 4 weeks, and the normal and model groups were fed the same volume of saline *via* intragastric administration. Echocardiographic studies were performed during the adaption week, before treatment, and after treatment. LVEF was calculated to assess left ventricular systolic function and cardiac function.

### Sample collection

At the end of the experiment, all rats were anesthetized with sodium pentobarbital. Blood was taken from the main abdominal vein into a plasma separator tube, and the samples were centrifuged at 4,000 rpm for 30 min at 4°C. The samples were stored at −80°C until use. The entire intestines were dissected with a sterile scalpel, and the contents of the intestines were collected with sterile lyophilized tubes (100–200 mg/tube). Fresh stool samples from each group were immediately frozen in a liquid nitrogen tank and then stored at −80°C.

### DNA extraction and 16S rRNA sequencing

According to the instructions of the MN NucleoSpin 96 Soi DNA extraction kit, the total genomic DNA was extracted from the samples. 16S rRNA sequencing was performed at the Beijing BMC Biotech Co., Ltd., (Beijing, China). The V3–V4 region of 16S rRNA genes was analyzed. The specific primers 338F (5'-ACTCCTACGGGAGGCAGCAG-3') and 806 R (5'-GGACTACHVGGGTWTCTAAT-3') with the barcodes were applied to amplify the 16S rRNA genes. The PCR mixture contains 5 μl of the purified product of PCR of the target region, MPPI-a of 2.5 μl, MPPI-b of 2.5 μl, and 2 × Q5 HF MM of 10 μl. The PCR products were monitored with 1.8% agarose gel, purified using the OMEGA DNA purification column, and then sequenced on an Illumina Miseq PE 3,000 platform.

### Non-targeted metabolomic analysis

Blood samples were centrifuged at 4,000 rpm for 30 min at 4°C to obtain plasma samples. An aliquot of the plasma sample (100 μl) was mixed with 300 μl of methanol containing 1 ppm of 2-chlorophenylalanine, vortexed for 2 min, and incubated at −20°C for 30 min. The mixture was centrifu, ged at 12,000 rpm for 10 min at 4°C, and the supernatant was obtained for further analysis.

Sample extracts were analyzed using an LC–QTOF–MS/MS. The analytical conditions for the system were as follows: an Aglient 1,290 ultra-high performance liquid chromatography (UPLC) (Agilent Technologies, Inc., USA) connected to an Aglient 6,545 quadrupole time-of-flight (QTOF) mass spectrometer. The chromatographic separation was achieved on an ACQUITY UPLC HSS T3 C18 column (1.8 μm, 2.1 × 100 mm) at 40°C using a mobile phase of 0.1% formic acid (A) and acetonitrile (B). Elution gradients used were as follows: 0–11 min, 95% A; 11–12 min, 10% A; 12–12.1 min, 10% A; and 12.2–14 min, 95% A. The flow rate was 0.40 ml/min and the injection volume was 2 μl. The mass spectrometry was performed in positive and negative ion modes. The parameters of the heated electrospray ionization method were as follows: sheath flow rate of 11 L/min, gas flow rate of 8 L/min, spray voltage of 250 V, positive and negative ionization, fragmentation voltage of 135 V, gas temperature of 325°C, sheath temperature of 325°C, and nebulizer pressure of 40 psi.

### Bioinformatic analysis and statistical analysis

The raw data were quality filtered using Trimmomatic (Version 0.33) (27), the primer sequences were identified and removed using Cutadapt (Version 1.9.1), followed by double-ended reads splicing and chimeric UCHIME (Version 8.1) removal using USEARCH (Version 10.0) (28), resulting in high-quality sequences for subsequent analysis. The sequences were clustered at a 97% similarity level (default) using USEARCH. Operational taxonomic units (OTUs) were filtered by default with a threshold of 0.005% of the number of all sequences sequenced. Raw data were uploaded to NCBI (ID: SUB12078416). The OTU sequences were obtained by splicing the filtered sequencing data with Qiime 2 software, and the abundance of each OTU was calculated and normalized for alpha analysis. Beta analysis was performed using the “FactoMineR” package in R software (Version 4.2.0), and a linear discriminant analysis was performed using the “lefseR” package (alpha analysis was performed using GraphPadPrism9 for statistical analysis and graphing, and linear discriminant analysis (linear discriminant analysis effect size, LEfSe) was performed using Mothur software and LEfSe software for detecting species differences between groups). Species with linear discriminant analysis (LDA) values >4 were considered to have statistically significant differences between groups.

Statistical analysis was performed in R. First, partial least squares discriminant (PLS-DA) analysis was performed using the “mixOmics” package and the “ropls” package, and model substitution tests were performed to screen out metabolites with intra-group differences from the metabolite summary table. The metabolites with RSD of ≤ 10% were subjected to Mfuzz clustering analysis (29). The subsequent analysis was mainly carried out around the metabolite clusters whose contents were shown trend of first rising and then falling or first falling and then rising in Control, Model and JSP group. These metabolites could be potential biomarkers for the treatment of heart failure.

### Weighted gene co-expression network analysis (WGCNA)

Operational taxonomic units (OTUs) of the intestinal microbiome were divided into modules by using weighted gene co-expression network analysis (WGCNA), then correlated with the metabolome to find metabolites and microbes that performed key functions, identified potential mechanisms involved in specific biological processes, and explored candidate biomarkers (30).

## Results

### Establishment of chronic heart failure model in SD rats

**Figure 1:**
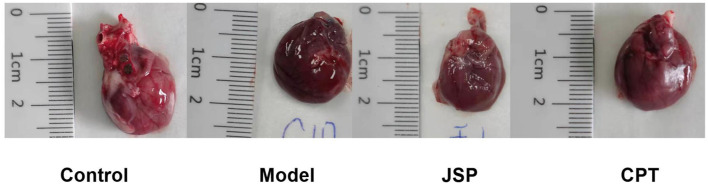
Representative images of the gross appearance of rat hearts.

After the model rats created by the left anterior descending coronary artery ligation for 4 weeks, the left ventricular of them were dilated and contractility was diminished. Rats showed symptoms of heart failure, such as hair and weight loss, feces rarefaction, reduction in activities, accidie, and extrados. The heart samples of each group were obtained by dissection, and the appearance is shown in Figure 1.

The left ventricular ejection fraction (LVEF) of the control group, the model group, and the JSP gastric perfusion group are shown in Table 1. Before being treated with given JSP and CPT, the LVEF of rats in the model group was significantly lower than that in the control group but there was no significant difference among rats in the model group, the JSP group, and the CPT group. After giving JSP and CPT to treatment groups for 4 weeks, the LVEF of rats in the model group was not changed significantly compared to the data from 4 weeks ago, but those in JSP and CPT were higher than that before. The LVEF of the JSP group and the CPT group had no significant difference but were significantly higher than that of the model group. Therefore, as can be seen from this indicator, both JSP and CPT had a good effect on heart failure. Then, the intestinal contents of each treatment group rat were obtained on a sterile bench to study their intestinal microbiome.

**Table 1:** The LVEF of rats in different groups (mean ± SD, *n* = 6).

**Group**	**Control**	**Model**	**Model + JSP**	**Model + CPT**
LVEF (Week 4)	80.12 ± 4.75a	56.53 ± 1.34b	57.07 ± 3.05b	56.65 ± 2.75b
LVEF (Week 8)	75.39 ± 4.18a	56.82 ± 2.06b	74.13 ± 1.69a	75.13 ± 1.76a

### Diversity analysis of intestinal microbiome

**Figure 2:**
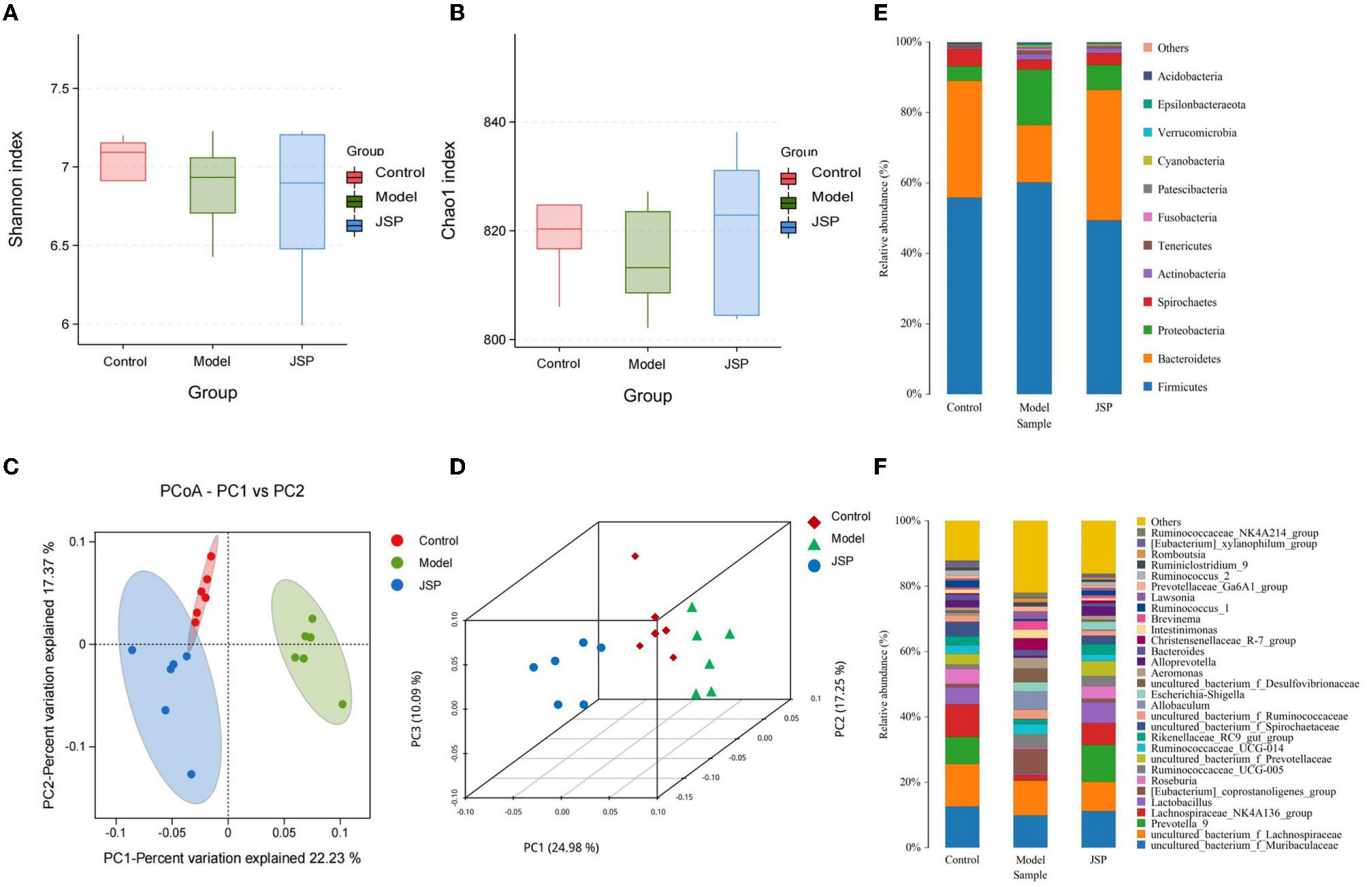
The structure of the intestinal microbiome in the control, model, and JSP groups. **(A, B)** Shannon and Chao1 indices of α diversity. **(C, D)** Analysis of β diversity of intestinal microbiome in each group's rats. PCoA analysis of intestinal microbiome based on the OTU data of the control, model, and JPS groups. Each point represents a sample. A clear separation is observed between the samples of control (*n* = 6), model (*n* = 6), and JPS (*n* = 6) groups. **(E, F)** Percentage of total bacteria presented at phyla and genus levels, respectively.

After extracting the DNA from the intestinal microbiome of the rats in each treatment group, their 16S rRNA sequence data were determined by high-throughput sequencing. After filtering and splicing these sequencing data, the sequence and abundance of 889 OTUs were obtained. The normalized abundance of OTUs was used to perform intestinal microbiota diversity analysis. In the alpha diversity-related indicators shown in Figures 2A, B, the Chao1 index and Shannon index were not significantly different (*P* > 0.05) in the species richness and evenness of each group. The between-group variability analysis of the four diversity indexes is detailed in Supplementary Table 1. It can be seen that there was no significant difference in the diversity of gut microbiota species between HF rats and normal rats.

The results of the β diversity analysis (Figures 2C, D), based on the principal coordinate analysis of relative abundance (PCoA), showed that the community structures of intestinal microbiome were different among the control group, the model group, and the JSP group. According to the PERMANOVA analysis, the variance contributions to the difference in the bacterial structure were 10.09 and 24.98%. The intestinal microbiome structures of rats in each treatment group were significantly different, and the *Pr*-value (>F) was < 0.001.

The phylum-level species of gut microbes in this study are shown in Figure 2E. The largest average proportion of each bacterial community is Firmicutes, which accounts for 40–60%. Compared with the control group, the Bacteroidetes proportion was decreased in the model group and then increased in the JSP group. However, the proportion of Proteobacteria in the same treatment showed the opposite trend of change.

The genus-level species of gut microbes in this study are shown in Figure 2F. Compared with the control group and the JSP group, the proportions of *Prevotella_9, Lactobacillus*, and *Lachnospiraceae_NK4A136_group* were decreased in the model group, whereas the proportion of *Allobaculum* in the model group was increased compared to that in the control and the model groups.

### Analysis of intestinal microbiome differences

**Figure 3:**
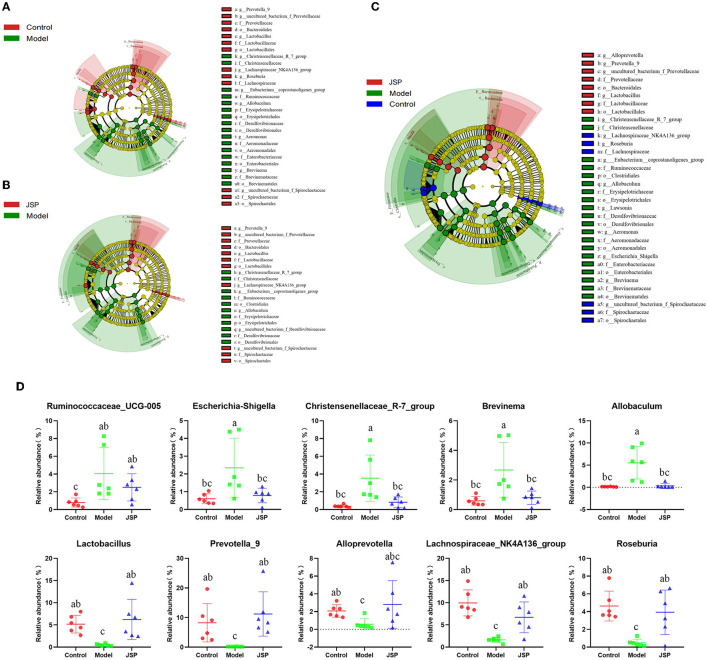
LEfSe analysis for the gut microbiota alterations in different groups. **(A, B)** Microbial signatures for the model vs. control groups and JSP vs. model groups, respectively. **(C)** Overall gut microbiota community with different abundances. **(D)** Identified gut microbes with significant differences between groups. Data are shown as the means ± SEM (*n* = 6). Different lowercase letters indicated in the figure depict significant between-group variation (*P* < 0.05).

Linear discriminant analysis effect size (LEfSe) was used to identify similar and dominant microbial species in the gut of rats in each treatment group. Compared with the control group, the abundance of Proteobacteria and their derivatives taxa were significantly higher in the model group (Figure 3A). The intestinal microorganisms of rats in the JSP group were rich in Bacteroides and their derivative taxa, including Bacteroidia, Bacteroidales, Prevotella, and Alloprevotella (Figure 3B). In addition, the results of LEfSe also indicated that the floras of Proteobacteria, Erysipelotrichia, and Gammaproteobacteria were key to the isolation of the model group from the other two groups (Figure 3C). The relative abundances of key differentiated bacterial genes in different groups are summarized in Figure 3D.

### Determination of rat plasma metabolites and their function

Diseases and drugs often affect the intestinal microbiome, causing changes in their metabolites, which enter the bloodstream through the intestines and have a positive or negative effect on the host's physiology. Therefore, metabolomic analysis of plasma samples from rats of different treatment groups was performed by high-performance liquid chromatography-mass spectrometry (HPLC-MS) in this study. There were 6,565 and 9,203 peaks in total that were identified in negative and positive ion modes, respectively. Next, the peaks were clustered using partial least squares discriminant analysis (PLS-DA) to obtain more reliable metabolites with significant differences between treatment groups and to further test the validity of the method.

**Figure 4:**
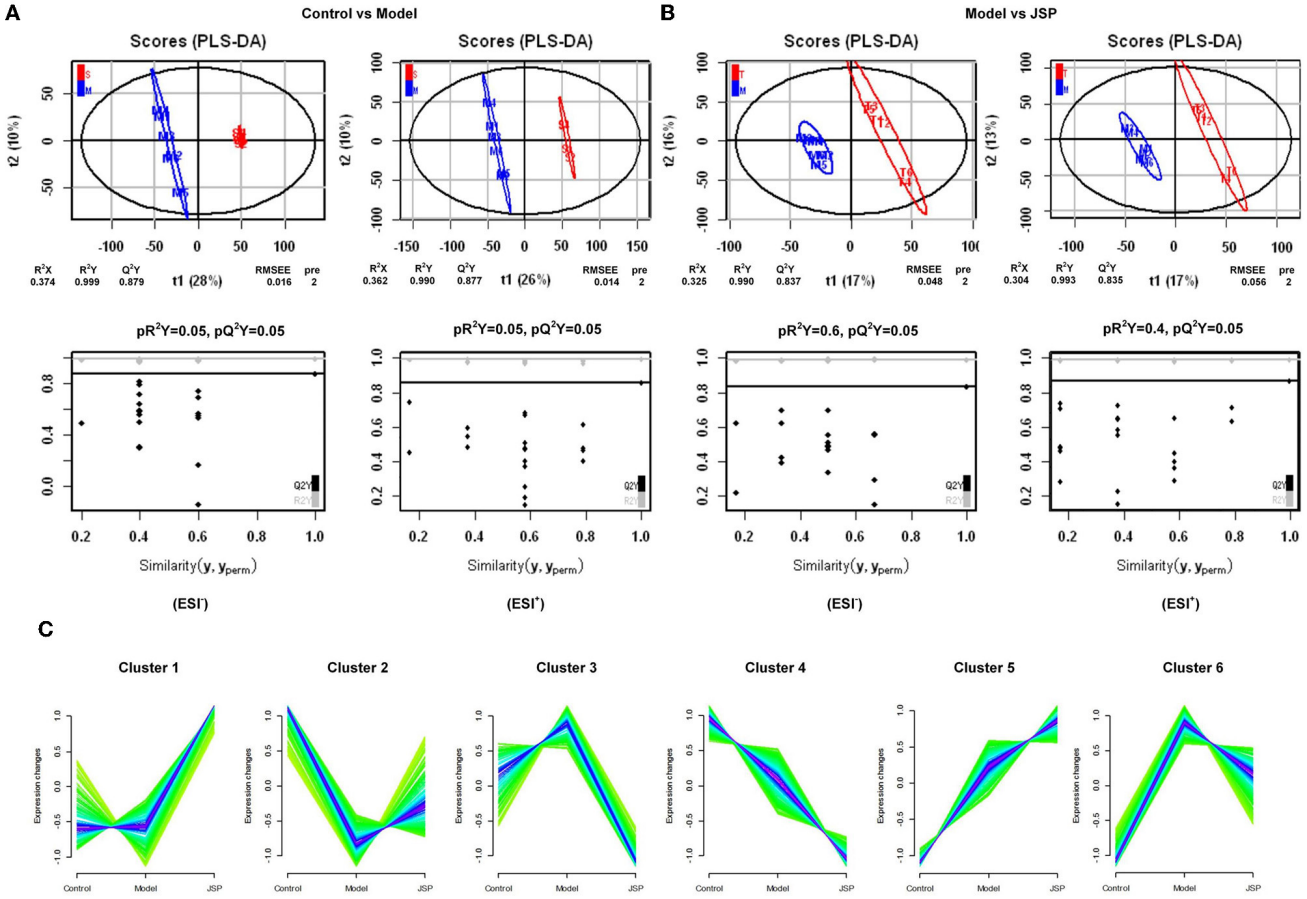
The significant changes in plasma metabolic profiles in HF and treated rats. **(A, B)** Plots of PLS-DA scores of all peak features in positive and negative ion mode from the untargeted metabolomics analysis of plasma samples of the rats in control vs. model and model vs. JSP, respectively. **(C)** Clustering diagram of relative abundance patterns of the plasma metabolites based on the Mfuzz algorithm.

The results showed that the plasma metabolism data clusters under different treatments were separated from each other, indicating the presence of many different potential biomarkers (Figures 4A, B). The interpreted and validated values for the differences in the model were R^2^X = 0.374, R^2^Y = 0.999, Q^2^Y = 0.879 (control group vs. model group) and R^2^X = 0.325, R^2^Y = 0.990, Q^2^Y = 0.837 (control group vs. JSP group) in negative ion mode, and R^2^X = 0.362, R^2^Y = 0.999, Q^2^Y = 0.877 (control group vs. model group) and R^2^X = 0.304, R^2^Y = 0.993, Q^2^Y = 0.835 (control group vs. JSP group) in positive ion mode. This indicated that the model adequately explains the source of differences between the samples in each treatment group.

Many metabolites were identified by analyzing the peaks on the tandem mass spectrometry, combining the accurate relative molecular weight, and obtaining the structural information from the compound structure database. Based on the analysis method of fuzzy C-means clustering, we carried out the clustering analysis of metabolites whose relative average deviation within the selected group was < 10%. Therefore, these metabolites were clustered into six groups. The change trends of metabolite content in the same clusters were similar in different treatment groups. But there were significant differences in change trends among different cluster groups. As shown in Figure 4C, in Cluster 1, there was no change between control and model groups but was significantly increased in the JSP group. In Cluster 2, the metabolite content was the lowest in the model group. On the contrary, the metabolite content in Clusters 3 and 6 was the highest in the model group. The metabolite content of Clusters 4 and 5 also showed the opposite trend, which was gradually decreasing and gradually increasing in the control, model, and JSP groups, respectively.

Based on the trend of these metabolite content, we speculated that the metabolites in Clusters 2, 3, and 6 were involved in improving the physiological process of heart failure. Therefore, the function of metabolites in these three clusters was performed by metabolic pathway enrichment analysis. Based on ConsensusPathDB (http://ConsensusPathDB.org) analysis, these metabolites were enriched in the following pathways: heme biosynthesis from uroporphyrinogen-III, heme biosynthesis II, pyrimidine deoxyribonucleotides biosynthesis I, M phase, degradation of cysteine and homocysteine heme biosynthesis, cell cycle, metabolism of nucleotides, metabolism of vitamins and cofactors, interconversion of nucleotide di- and triphosphates, sulfur amino acid metabolism, tryptophan metabolism, ferroptosis, and inflammatory mediator regulation of TRP channels (Table 2). At the same time, eight key metabolites were obtained, including ferrous ion (C14818), protoporphyrin IX (C01079), dihydrofolic acid (C00415), deoxyuridine-5'-triphosphate (C00460), nicotinamide (C00153), 3-mercapto-2-oxopropionic acid (C00957), propionylcarnitine (C03017), acyclovir (C06810), and their contents in the control and JSP groups were higher than that in model group. There are seven metabolites that were reduced in the control and JSP groups: kynurenic acid (C01717), reduced L-glutathione (C00051), 5-hydroxyindolepyruvate (C05646), 2-oxohexanedioic acid (C00322), 1-stearoyl-2-hydroxy-sn- glycero-3-phosphoethanolamine (C21484), cinnamaldehyde (C00903), and icilin (C20171) (Table 2).

**Table 2:** Metabolic pathway of plasma metabolite enrichment in groups 2, 3, and 6.

**Pathway**	**Pathway source**	**Overlapping metabolites**	**NO. of all pathway metabolites**	***p*-value**	***q*-value**	**Cluster**
Biosynthesis from uroporphyrinogen-III I	MouseCyc	C01079; C14818	11 (11)	0.001	0.012	2
Biosynthesis II	MouseCyc	C01079; C14818	18 (18)	0.001	0.012	2
Pyrimidine deoxyribonucleotides *de novo* biosynthesis I	MouseCyc	C00415; C00460	20 (20)	0.002	0.012	2
M Phase	Reactome	C14818; C00153	22 (25)	0.002	0.012	2
Degradation of cysteine and homocysteine	Reactome	C14818; C00957	24 (30)	0.002	0.012	2
Biosynthesis	Reactome	C01079; C14818	27 (33)	0.003	0.013	2
Cell Cycle, Mitotic	Reactome	C14818; C00153	34 (39)	0.005	0.014	2
Peroxisomal lipid metabolism	Reactome	C14818; C03017	35 (59)	0.005	0.014	2
Metabolism of porphyrins	Reactome	C01079; C14818	36 (42)	0.006	0.014	2
DNA Repair	Reactome	C14818; C00153	37 (51)	0.006	0.014	2
Cell Cycle	Reactome	C14818; C00153	38 (43)	0.006	0.014	2
Metabolism of nucleotides	Reactome	C14818; C00460; C00415	128 (148)	0.007	0.015	2
Metabolism of vitamins and cofactors	Reactome	C14818; C00153; C00415	132 (172)	0.007	0.015	2
Interconversion of nucleotide di- and triphosphates	Reactome	C00415; C00460	46 (50)	0.009	0.016	2
Sulfur amino acid metabolism	Reactome	C14818; C00957	46 (57)	0.009	0.016	2
Tryptophan metabolism–Mus musculus (mouse)	KEGG	C05646; C00322; C01717	83 (83)	0.003	0.034	3
Ferroptosis–Mus musculus (mouse)	KEGG	C00051; C21484	31 (31)	0.005	0.034	3
Inflammatory mediator regulation of TRP channels–Mus musculus (mouse)	KEGG	C20171; C00903	35 (35)	0.005	0.042	6

### Intestinal microbiome combined with key metabolites WGCNA analysis

**Figure 5:**
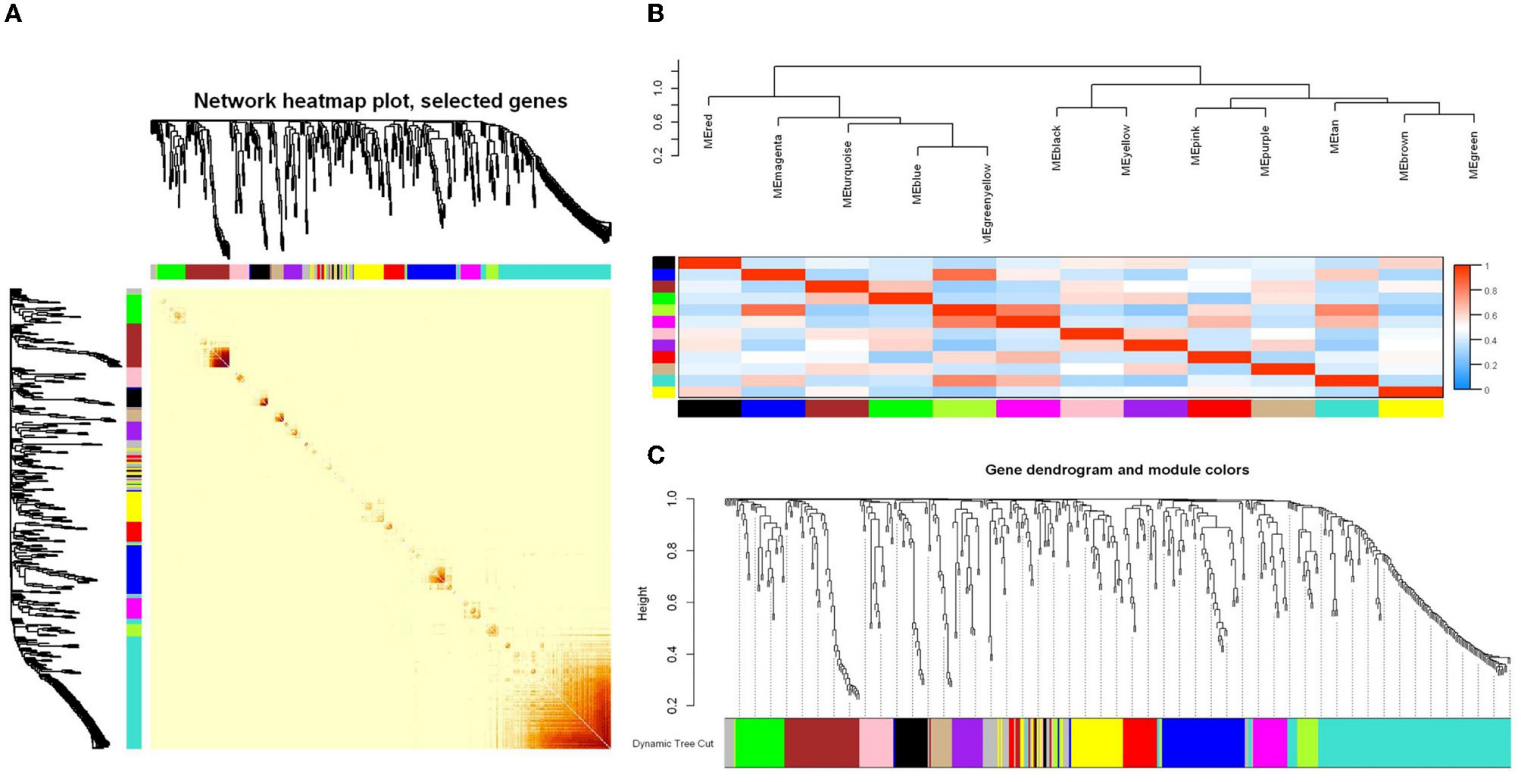
WGCNA analysis of the intestinal microbiome co-expression network based on the relative abundance of OTUs. **(A)** Relative abundance correlation heat map of intestinal microbiome OTUs. **(B)** Connectivity and cluster analysis of OTUs' relative abundance in a different module, and the heatmap of connectivity of them. **(C)** Clustering dendrogram of OTUs, with dissimilarity based on the topological overlap, together with assigned module colors. The clustered branches represent different modules, and each line represents one OTU.

In this study, the WGCNA package in R software was used to construct a co-expression network. From the results of 16S rRNA sequencing of the intestinal microbiota of 16 rats, 483 OTUs with average relative abundance >1 and genus-level information were selected to construct the co-expression network. The data obtained earlier were used to construct the scale-free network. First, the adjacency matrix was calculated based on the expression value matrix, and then the topological overlap matrix reflected the similarity of the common expressions was derived. Based on the scale-free topology with R^2^ = 0.85, the Pearson correlation matrix of the 16S rRNA region OTUs was transformed into a strengthened adjacency matrix according to the power of r = 11 and r = 6, respectively. Then, the topological overlap matrix was used for hierarchical clustering to draw clustering trees that could jointly characterize the overall distribution of similarity. Finally, the generated clustering trees were cut by the dynamic cut tree algorithm. In this process, OTUs with high similarity in the common expression were clustered into the same branch, and different branches of the clustering tree represented different modules, each of which was assigned a specific color (Figures 5A–C). After constructing the co-expression network, 13 co-expression modules were obtained for intestinal bacterial OTUs (Table 3). Among them, the MEturquoise module had the highest number of OTUs with 128, while the MEtan module had the lowest number of OTUs with 13.

**Table 3:** Number of OTUs in each module.

**Modules**	**OTU-number**
Black	23
Blue	54
Brown	52
Green	30
Greenyellow	19
Gray	36
Magenta	21
Pink	23
Purple	19
Red	25
Tan	13
Turquoise	128
Yellow	40

**Figure 6:**
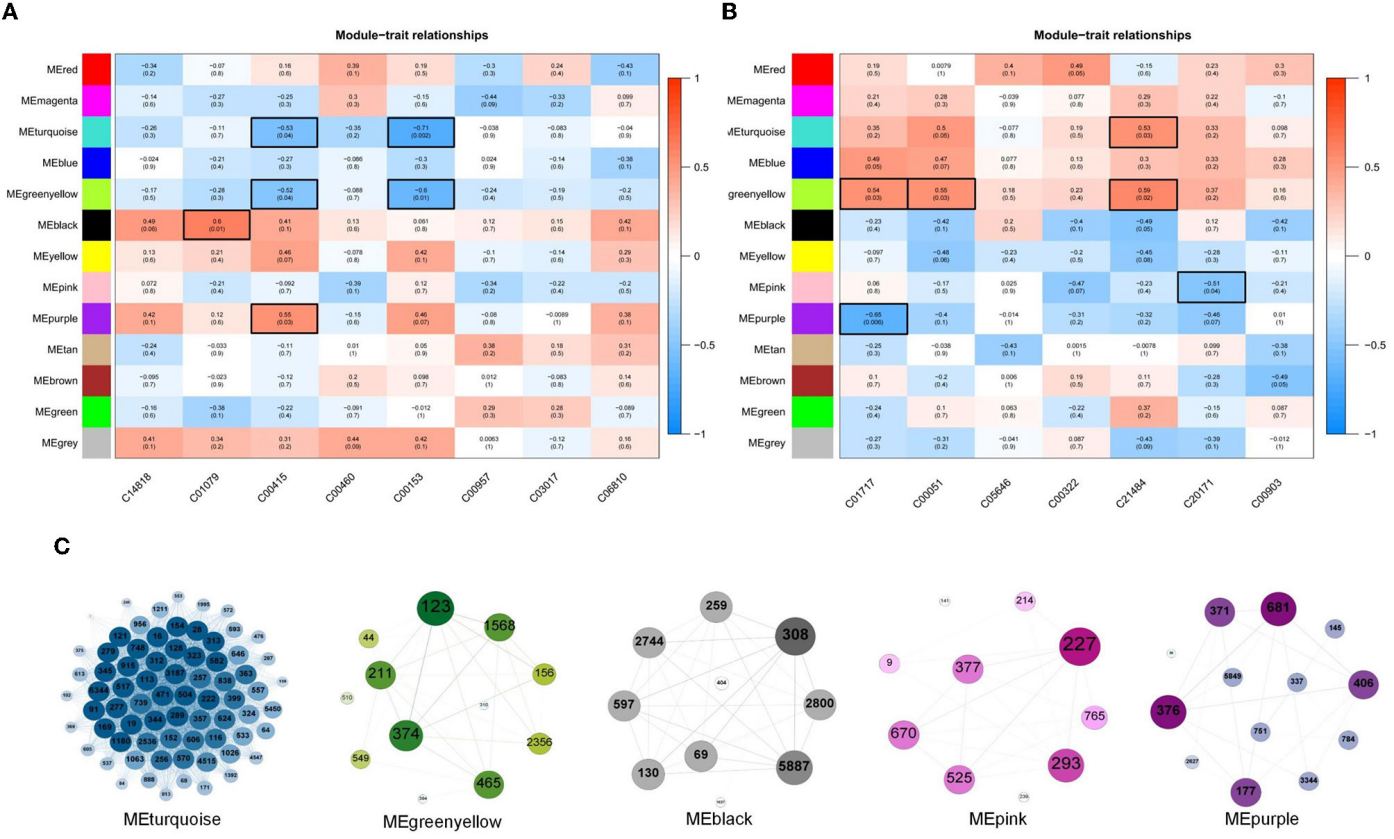
**(A, B)** Associations of different color modules and plasma metabolite from Clusters 2, 3, and 6. In the heatmap, each row corresponds to a module eigengene (ME) and each column to a trait. Each cell contains the corresponding correlation and *p*-value. The tables were color-coded by correlation according to the color legend. **(C)** Weighted correlation network of OTUs in five ME modules, including MEturquoise, MEgreenyellow, MEblack, MEpink, and MEpurple.

The results of the correlation analysis of metabolite content and the relative abundance of OTUs in Clusters 2, 3, and 6 are shown in Figures 6A, B, respectively. Evidently, as shown in Figure 6A, the content of C01079 and C00415 had significant positive correlations with MEblack and MEpurple modules, respectively. The content of C00415 and C00153 had significant negative correlations with MEturquoise and MEgreenyellow modules, respectively. As shown in Figure 6B, the content of C01717, C00051, and C21484 had significant positive correlations with MEturquoise and MEgreenyellow modules, respectively. The content of C01717 and C20171 had significant negative correlations with MEpink and MEpurple, respectively. The OTUs in the five modules were separately constructed as correlation networks, and the results are shown in Figure 6C. The number in each ball was the ID number of the OTU, as detailed in Supplementary Table 1.

**Figure 7:**
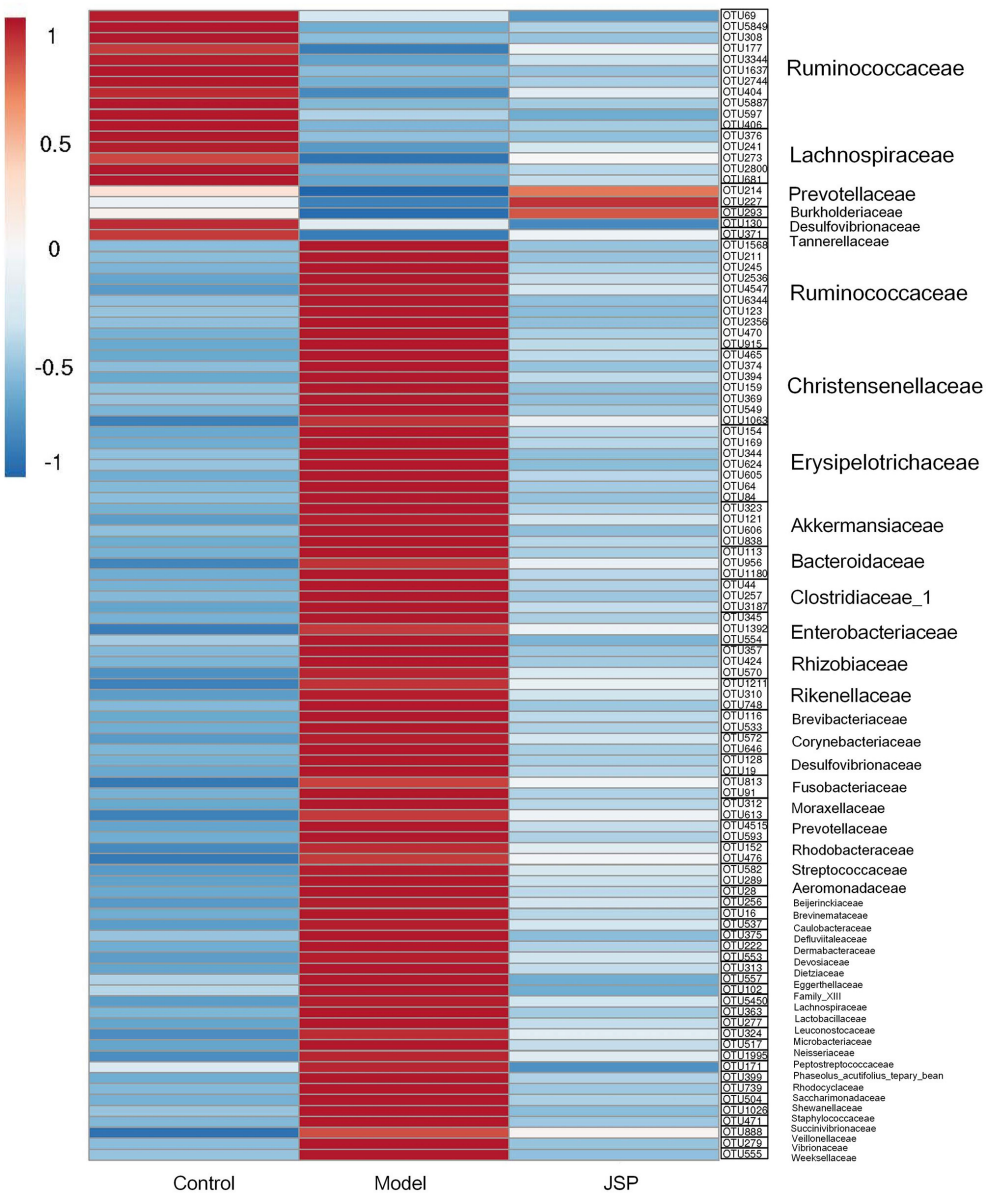
The heatmap of intestinal microbiome OTUs related to selected plasma metabolites from Clusters 2, 3, and 6.

As shown in Figure 7, the heat map was constructed by the mean relative abundance of key flora OTUs identified from WGCNA analysis, in which the family and genus information of these OTUs are classified, as shown in Figure 7. Overall, these bacteria were mainly distributed in six families, which were Ruminococcaceae, Chritensenellaceae, Erysipelotrichaceae, Akkermansiaceae, Clostridiaceae-1 and Lachnospiraceae (Figure 7). After correlation analysis of seven key metabolites and identified OTUs (Supplementary Figures 2, 3), it was found that OTU1637, OTU2744, OTU404, OTU5887, and OTU597 at *Ruminococcaceae_UCG-014* genus, and had a significantly positive correlation with C01079 content. C00153 and C00415 contents had a significantly negative correlation with OTU2536, OTU4547, OTU6344, OTU915, OTU1568, OTU1063, OTU159, OTU369, OTU374, OTU394, OTU465, and OTU549, which belonged to *Ruminococcaceae_UCG-005, Christensenellaceae_R-7_group*, and *Erysipelotrichaceae*. However, C21484 content had a significantly positive correlation with OTU1568, OTU2536, OTU4547, OTU6344, OTU374, OTU394, OTU465, OTU549, OTU159, and OTU369 at the three genera mentioned above. C01717 content had a significantly positive correlation with OTU177, OTU3344, OTU406, and OTU5849 which belonged to Ruminococcaceae (Supplementary Table 1).

## Discussion

Mounting pieces of evidence have revealed an association between microbial composition and their metabolites in HF. Recent studies had focused on HF with changes in intestinal microbiome structure but ignored its connection with microbial metabolites. Thus, a systematic investigation of the relevance between the intestinal microbiome and its metabolites is greatly warranted. We valued the changing intestinal microbiome and potential metabolites associated with the development and prognosis of HF. Through clustering and metabolite pathway analysis, 15 metabolites with clear physiological functions were identified in the control, the heart failure model, and the JSP treatment group, using the pathways of biosynthesis from uroporphyrinogen-III, biosynthesis II, pyrimidine deoxyribonucleotides biosynthesis I, degradation of cysteine and homocysteine, metabolism of nucleotides, and metabolism of vitamins and cofactors. After combinedly analyzing the contents of these metabolites and the intestinal microorganism OTUs by WGCNA, 116 bacteria that were significantly related to the content change of these metabolites were identified. These associations highlighted potential interactions of microbe and metabolites, which helped to further reveal the effect process by JSP *in vivo* during the development and prognosis of HF.

There are no significant differences in intestinal microbial diversity among Control, Model, and JSP groups. We have identified some bacteria altered by JSP treatment through LEfSe analysis, such as Erysipelotrichia, Anaplasma, and Gammaproteobacteria. It was found that they were the key differentiators that distinguished the model group from the control and the JSP treatment groups (Figure 3D). The changes in the abundance or ratio of several bacteria could indicate whether the intestinal microbiome was in disorder. For instance, the ratio of Firmicutes to Bacteroides (F/B) was an important indicator of intestinal microbiome disorder (31). Compared with healthy people, F/B in the intestinal microbiome of patients with hypertension and heart failure was increased (32).

Among them, the bacteria of Erysipelotrichia under Firmicutes had the function of increasing the permeability of the intestinal mucosa and mediating the inflammatory response (33). In this study, JSP may repair the intestinal barrier by inhibiting the F/B ratio to promote HF prognosis. Some bacteria contained in *Lachnospiraceae_NK4A136_group* (34, 35), *Alloprevotella* (36), and *Roseburia* (37, 38) could produce some short-chain fatty acids in the host intestine to regulate colon movement, immune maintenance, and anti-inflammatory, which may be related to protection from the host's stress reaction, and increased continuously in the prognosis of HF. It was shown that serum TMAO levels were positively associated with an increased abundance of *Ruminococcaceae_UCG_005 and Christensenellaceae_R-7_group* (39). TMAO is a molecular metabolite derived from the gut microbiota, which may directly affect the heart by inducing myocardial hypertrophy and fibrosis, endothelial cell and vascular inflammation, as well as cardiac mitochondrial dysfunction, thereby aggravating the progress of HF (40–42). *Prevotella-9* was negatively correlated with cardiac ejection fraction in rats with spontaneous hypertensive HF (43). In this study, we found that the abundance of several bacteria in Prevotella-9 increased after JSP treatment. We speculated that its function was related to improving the ejection fraction, but the specific mechanism was not clear.

**Figure 8:**
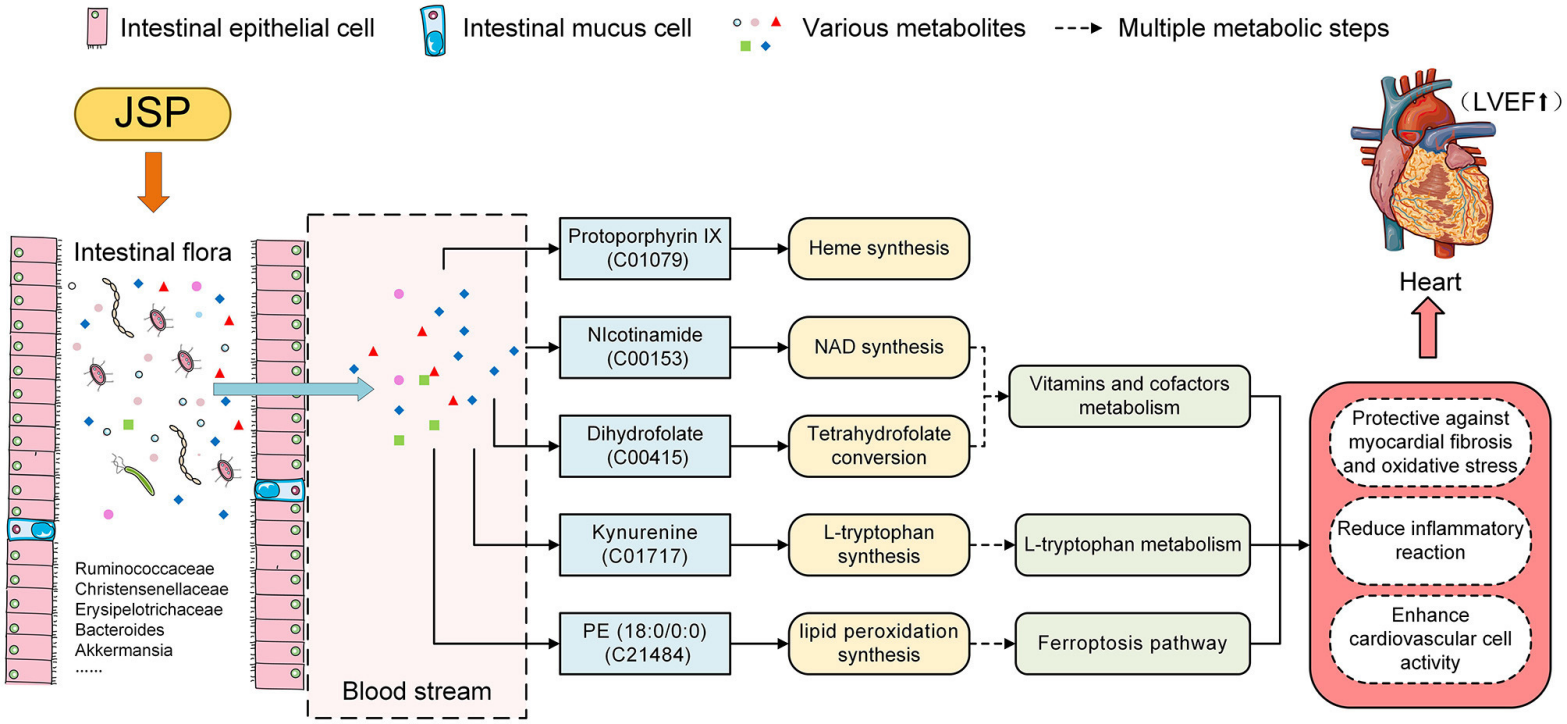
JSP modified heart failure by the effect on the intestinal microbiome and metabolites in the blood.

To identify plasma metabolites in response to JSP gavage, we attempted to analyze the changes in the content of these metabolites in different treatment groups using fuzzy c-means clustering. Three change clustering modes that respond to JSP processing have been selected, namely Clusters 2, 3, and 6 (Figure 4C). Through the functional analysis of metabolites in these groups, seven metabolites were found, which were distributed in six pathways related to the development and prognosis of HF (Table 1). First, protoporphyrin IX (C01079) is the intermediate product of heme biosynthesis, which is a key metabolite used to bind oxygen and oxidize the guanidine nitrogen of L-arginine to form nitric oxide (NO), playing a role as a vasodilator, as well as the citrulline under the action of nitric oxide synthase (44). The anti-inflammatory carbon monoxide and bilirubin are produced by processes of heme metabolism, which further enhances the anti-inflammatory effect and ultimately relieves atherosclerosis (45). Heme is also an important component of antioxidant functional proteins and enzymes in cardiomyocytes (46). Heme has been shown to be protective against myocardial fibrosis and oxidative stress through inducting of heme oxygenase 1 and the activation of the phosphatidylinositol 3-kinase/AKT signaling pathway (47). In this study, there were 10 bacteria that were significantly related to protoporphyrin IX, which belong to *Ruminococcaceae_UCG-014, Ruminiclostridium_6, Fournierella, Eubacterium xylanophilum_group, Roseburia*, and *Desulfovibrio*. These bacteria may participate in the synthesis of protoporphyrin IX and indirectly affect the change of heme content under the influence of JSP, which could enhance the antioxidant function of myocardial cells, reduce the inflammatory reaction, and ultimately improve the effectiveness of heart failure (Figure 8).

Then, in the metabolism pathway of vitamins and cofactors, nicotinamide (C00153) is the precursor of nicotinamide adenine dinucleotide (NAD), which is participated in the wide range of reactions, including regulation of cellular redox status, energy metabolism, and mitochondrial biogenesis (48). In several models of heart failure, myocardial NAD levels have been depressed disturbed mitochondrial function remodeled metabolism, and occurred inflammation. The emerging evidence has suggested that regulating NAD homeostasis by NAD precursor supplementation has therapeutic efficiency in improving myocardial bioenergetics and function (49). Under JSP treatment, the abundance of microbiota under 57 genera in rat intestines, including *Ruminococcaceae_UCG-005, Christensenellaceae_R-7_group, Akkermansia*, and *Bacteroides*, was significantly correlated with nicotinamide. These bacteria may directly or indirectly participate in the synthesis of nicotinamide so that it could achieve the purpose of alleviating heart failure symptoms (Figure 8). Dihydrofolate (C00415) is an intermediate for the conversion of folic acid to tetrahydrofolic acid, which is a member of the B-vitamin family, and is essential for amino acid metabolism (50). Folic acid and its active metabolite 5-methyl tetrahydrofolate improved nitric oxide (NO) bioavailability by increasing endothelial NO synthase coupling and NO production as well as by directly scavenging superoxide radicals. By improving NO bioavailability, folic acid may protect or improve endothelial function, thereby preventing or reversing the progression of CVD in those with overt disease or elevated CVD risk (51). After JSP gavage treatment, it was found that the abundance of 57 bacteria distributed in 29 families and 40 genera in the intestinal microbiome of rats was significantly correlated with the dihydrofolate content in plasma (Figure 8).

Dihydrofolate (C00415) and nicotinamide (C00153) are vitamin B derivatives, which are closely related to a variety of intestinal microbiomes (52, 53) and the development process of heart failure. In these intestinal microflorae related to dihydrofolate (C00415), the secondary metabolites produced by the degradation of *Ruminococcaceae_UCG-014* need to be degraded by Bacteroidetes (54). As the main component of human and animal intestinal microflora, the normal growth of these bacteria depended on porphyrins, including protoporphyrin IX or heme with iron (55) (Figure 8).

Kynurenine (C01717), a metabolite of the L-tryptophan pathway, may mediate immunomodulation, oxidant defense, and apoptosis (56), which are considered pathogenic features in the development of heart failure, and has been shown to predict cardiovascular events (57, 58). Similarly, PE (18:0/0:0) (C21484) participated in the process of lipid peroxidation (LPO) in the pathway of ferroptosis. LPO which is one of their metabolites is involved in immune responses and cell deaths (58, 59). Kynurenine and PE (18:0/0:0) were significantly correlated with the abundance of 10 and 49 species of bacteria, respectively, in this research. It is speculated that these bacteria may, under the influence of JSP, regulate these two metabolites' contents directly or indirectly to alleviate the course of cardiovascular disease and increase the antioxidant capacity of cardiovascular cells (Figure 8).

Results of intestinal microbiome analysis revealed that JSP not only adjusted gut microbiota disturbances by enriching species diversity, reducing the abundance of pathogenic bacteria, such as *Allobaculum*, and *Brevinema*, as well as increasing the abundance of beneficial bacteria, including *Lactobacillus*, and *Lachnospiraceae_NK4A136_group* but also improved metabolic disorders by reversing metabolite plasma levels to normality. The results of the correlation analysis demonstrated a significant association between intestinal microbiota and plasma metabolic profile. The key relationships in our research would illustrate the underlying mechanism of JSP to treat heart failure by affecting intestinal microbiome and plasma metabolites as well as provide evidence for the interpretation of the mechanism in HF. But the physiological mechanism of these bacteria with the potential function of improving heart failure and how to regulate these endogenous metabolites are still unclear. In the future, we will conduct in-depth research on the function of these intestinal microbiomes to further improve the mechanism of JSP in treating heart failure.

## Data availability statement

The datasets presented in this study can be found in online repositories. The names of the repository/repositories and accession number(s) can be found below: https://www.ncbi.nlm.nih.gov/, BioProject ID PRJNA881788.

## Ethics statement

The animal study was reviewed and approved by the Research Ethical Committee of Guangdong Pharmaceutical University (Guangzhou, China).

## Author contributions

XHe, PL, and JM contributed to the design concepts of this whole study. XC carried out the animal experiment, performed data analyses, and drafted the manuscript. YS, XHu, and JC carried out the animal experiment and collected data. All authors have read and approved the content of the manuscript.

## Supplementary material

Supplementary Figure 1 The percentage of total bacteria presented at phylum **(A)**, class **(B)**, order **(C)**, family **(D)**, and genus **(E)** levels.

Supplementary Figure 2 The heatmap of intestinal microbiome OTUs related to selected plasma metabolites from Clusters 3 and 6.

Supplementary Figure 3 The heatmap of intestinal microbiome OTUs related to selected plasma metabolites from Cluster 2.

## References

[1] Rogers C Bush N. Heart failure pathophysiology, diagnosis, medical treatment guidelines, and nursing management. Nursing Clin North Am. (2015) 50:787–99. 10.1016/j.cnur.2015.07.012 26596665

[2] Dickstein K. Diagnosis and assessment of the heart failure patient: the cornerstone of effective management. Eur J Heart Fail. (2005) 7:303–8. 10.1016/j.ejheart.2005.01.003 15718169

[3] Lozupone CA Stombaugh JI Gordon JI Jansson JK Knight R. Diversity, stability and resilience of the human gut microbiota. Nature. (2012) 489:220–30. 10.1038/nature11550 22972295 PMC3577372

[4] Buglioni A Burnett JC. A gut-heart connection in cardiometabolic regulation. Nat Med. (2013) 19:534–6. 10.1038/nm.3196 23652101 PMC4512286

[5] Bartolomaeus H McParland V Wilck N. Gut-heart axis. How gut bacteria influence cardiovascular diseases. Herz. (2020) 45:134–41. 10.1007/s00059-020-04897-0 32077981

[6] Pasini E Aquilani R Testa C Baiardi P Angioletti S Boschi F. Pathogenic gut flora in patients with chronic heart failure. Jacc-Heart Failure. (2016) 4:220–7. 10.1016/j.jchf.2015.10.009 26682791

[7] Lin L. jie ZS, yuan HS, Bin C, Hong Q, xi HZ. Changes of gut microbiome composition and metabolites associated with hypertensive heart failure rats. BMC Microbiol. (2021) 21:141. 10.1186/s12866-021-02202-5 33952214 PMC8097775

[8] Zhang Y Zhang S Li B Luo Y Gong Y Jin X. Gut microbiota dysbiosis promotes age-related atrial fibrillation by lipopolysaccharide and glucose-induced activation of NLRP3-inflammasome. Cardiovasc Res. (2022) 118:785–97. 10.1093/cvr/cvab114 33757127

[9] Tracey GX Grace E. X HC, P BJ, V HJ, Venkatesh R, et al. Probiotic administration attenuates myocardial hypertrophy and heart failure after myocardial infarction in the rat Circulation. Heart Failure. (2014) 7:491–9. 10.1161/CIRCHEARTFAILURE.113.000978 24625365

[10] Anderson MK Ferranti EP Couvillon AE Emma M French EC Miller RC. The heart and gut relationship: a systematic review of the evaluation of the microbiome and trimethylamine-N-oxide (TMAO) in heart failure. Heart Fail Rev. (2022) 27:2223–49. 10.1007/s10741-022-10254-6 35726110

[11] Cristina M Massimo M Marina C Maria P. J BM, Jeff T, et al. Metabolomic study of carotid-femoral pulse-wave velocity in women. J Hypertension. (2015) 33:791–6. 10.1097/HJH.0000000000000467 25490711 PMC4354457

[12] Zhu M-j Wang Y-p Xie S-y Liu W-h Li B Wang Y-x. Protective effects of Jiashen Prescription on myocardial infarction in rats. Chinese J Integrative Med. (2015) 21:417. 10.1007/s11655-014-1751-4 24817316

[13] Sun N Zhang K Geng W Er X Gao W He Y. Analysis of chemical constituents of Jiashen Tablet extract by UPLC-Q-TOF-MS. Chinese Herbal Med. (2018) 49:293–304. 10.7501/j.issn.0253-2670.2018.02.006

[14] Cui L Wang Y Yu R Li B Xie S Gao Y. Jia-Shen decoction-medicated serum inhibits angiotensin-II induced cardiac fibroblast proliferation *via* the TGF-beta 1/Smad signaling pathway. Mol Med Rep. (2016) 14:1610–6. 10.3892/mmr.2016.5405 27315199 PMC4940101

[15] Feng W Ao H Peng C Yan D. Gut microbiota, a new frontier to understand traditional Chinese medicines. Pharmacol Res. (2019) 142:176–91. 10.1016/j.phrs.2019.02.024 30818043

[16] Lin L Luo L Zhong M Xie T Liu Y Li H. Gut microbiota: a new angle for traditional herbal medicine research. RSC Adv. (2019) 9:17457–72. 10.1039/C9RA01838G 35519900 PMC9064575

[17] Lin TL Lu CC Lai WF Wu TS Lu JJ Chen YM. Role of gut microbiota in identification of novel TCM-derived active metabolites. Protein Cell. (2021) 12:394–410. 10.1007/s13238-020-00784-w 32929698 PMC8106560

[18] Do MH Seo YS Park HY. Polysaccharides: bowel health and gut microbiota. Crit Rev Food Sci Nutr. (2021) 61:1212–24. 10.1080/10408398.2020.1755949 32319786

[19] Gong X Li X Bo A Shi RY Li QY Lei LJ. The interactions between gut microbiota and bioactive ingredients of traditional Chinese medicines: a review. Pharmacol Res. (2020) 157:104824. 10.1016/j.phrs.2020.104824 32344049

[20] Yin L Li XX Ghosh S Xie CM Chen J Huang H. Role of gut microbiota-derived metabolites on vascular calcification in CKD. J Cell Mol Med. (2021) 25:1332–41. 10.1111/jcmm.16230 33369187 PMC7875928

[21] Nie QX Chen HH Hu JL Fan ST Nie SP. Dietary compounds and traditional Chinese medicine ameliorate type 2 diabetes by modulating gut microbiota. Crit Rev Food Sci Nutr. (2019) 59:848–63. 10.1080/10408398.2018.1536646 30569745

[22] Lu YH Wan HF Wu YJ Yang JH Yu L He Y. Naoxintong capsule alternates gut microbiota and prevents hyperlipidemia in high-fat-diet fed rats. Front Pharmacol. (2022) 13:843409. 10.3389/fphar.2022.843409 35387330 PMC8978017

[23] Ide T Tsutsui H Hayashidani S Kang DC Suematsu N Nakamura K. Mitochondrial DNA damage and dysfunction associated with oxidative stress in failing hearts after myocardial infarction. Circ Res. (2001) 88:529–35. 10.1161/01.RES.88.5.529 11249877

[24] Miao XL Chen JP Su YY Luo JY He Y Ma J. Plasma metabolomic analysis reveals the therapeutic effects of Jiashen tablets on heart failure. Front Cardiovasc Med. (2022) 9:1047322. 10.3389/fcvm.2022.1047322 36561767 PMC9763324

[25] Ding L Su XX Zhang WH Xu YX Pan XF. Gene expressions underlying mishandled calcium clearance and elevated generation of reactive oxygen species in the coronary artery smooth muscle cells of chronic heart failure rats. Chinese Med J. (2017) 130:460–9. 10.4103/0366-6999.199825 28218221 PMC5324384

[26] Burrell LM. R C, A PP, P C, M TA, I JC. Validation of an echocardiographic assessment of cardiac function following moderate size myocardial infarction in the rat. Clin Exp Pharmacol Physiol. (1996) 23:570–2. 10.1111/j.1440-1681.1996.tb02782.x 8800587

[27] Bolger AM Lohse M Usadel B. Trimmomatic: a flexible trimmer for Illumina sequence data. Bioinformatics. (2014) 30:2114–20. 10.1093/bioinformatics/btu170 24695404 PMC4103590

[28] Yoon SH Ha SM Lim J Kwon S Chun J. A large-scale evaluation of algorithms to calculate average nucleotide identity. Antonie Van Leeuwenhoek. (2017) 110:1281–6. 10.1007/s10482-017-0844-4 28204908

[29] Futschik ME Carlisle B. Noise-robust soft clustering of gene expression time-course data. J Bioinform Comput Biol. (2005) 3:965–88. 10.1142/S0219720005001375 16078370

[30] Langfelder P Horvath S. WGCNA an R package for weighted correlation network analysis. BMC Bioinform. (2008) 9:559. 10.1186/1471-2105-9-559 19114008 PMC2631488

[31] Miao X Chen J Su Y Luo J He Y Ma J He X. Correlation between firmicutes, bacteroidetes and firmicutes/bacteroidetes ratio and lipid profile in severely obese women in Rio De Janeiro - Brazil. J Acad Nutr Dietetics. (2022) 9:122. 10.1016/j.jand.2022.06.054

[32] Marques FZ Nelson E Chu PY Horlock D Fiedler A Ziemann M. High-fiber diet and acetate supplementation change the gut microbiota and prevent the development of hypertension and heart failure in hypertensive mice. Circulation. (2017) 135:964–77. 10.1161/CIRCULATIONAHA.116.024545 27927713

[33] Pan MF Wan CX Xie Q Huang RH Tao XY Shah NP. Changes in gastric microbiota induced by Helicobacter pylori infection and preventive effects of Lactobacillus plantarum ZDY 2013 against such infection. J Dairy Sci. (2016) 99:970–81. 10.3168/jds.2015-10510 26709179

[34] Ma LY Ni YH Wang Z Tu WQ Ni LY Zhuge F. Spermidine improves gut barrier integrity and gut microbiota function in diet-induced obese mice. Gut Microbes. (2020) 12:1832857. 10.1080/19490976.2020.1832857 33151120 PMC7668533

[35] Du SB Zhou HH Wang PF Wang XP Xue ZP Li J. Modulation effects of danshen-honghua herb pair on gut microbiota of acute myocardial ischemia model rat. FEMS Microbiol Lett. (2022) 369:1–10. 10.1093/femsle/fnac036 35349671

[36] Li Y Su XH Gao Y Lv CX Gao ZW Liu YP. The potential role of the gut microbiota in modulating renal function in experimental diabetic nephropathy murine models established in same environment. Biochim Biophys Acta Mol Basis Dis. (2020) 1866:165764. 10.1016/j.bbadis.2020.165764 32169506

[37] Tamanai-Shacoori Z Smida I Bousarghin L Loreal O Meuric V Fong SB. et al. Roseburia spp: a marker of health? Future Microbiol. (2017) 12:157–70. 10.2217/fmb-2016-0130 28139139

[38] Nie K Ma KJ Luo WW Shen ZH Yang ZY Xiao MW. Roseburia intestinalis: a beneficial gut organism from the discoveries in genus and species. Front Cell Infect Microbiol. (2021) 11:757718. 10.3389/fcimb.2021.757718 34881193 PMC8647967

[39] Gao J Yan KT Wang JX Dou J Wang J Ren M. Gut microbial taxa as potential predictive biomarkers for acute coronary syndrome and post-STEMI cardiovascular events. Sci Rep. (2020) 10:26–39. 10.1038/s41598-020-59235-5 32060329 PMC7021689

[40] Li ZH Wu ZY Yan JY Liu HL Liu QC Deng Y. Gut microbe-derived metabolite trimethylamine N-oxide induces cardiac hypertrophy and fibrosis. Lab Invest. (2019) 99:346–57. 10.1038/s41374-018-0091-y 30068915

[41] Makrecka-Kuka M Volska K Antone U Vilskersts R Grinberga S Bandere D. Trimethylamine N-oxide impairs pyruvate and fatty acid oxidation in cardiac mitochondria. Toxicol Lett. (2017) 267:32–8. 10.1016/j.toxlet.2016.12.017 28049038

[42] Sun XL Jiao XF Ma YR Liu Y Zhang L He YZ. Trimethylamine N-oxide induces inflammation and endothelial dysfunction in human umbilical vein endothelial cells *via* activating ROS-TXNIP-NLRP3 inflammasome. Biochem Biophys Res Commun. (2016) 481:63–70. 10.1016/j.bbrc.2016.11.017 27833015

[43] Shao MM Zhu Y. Long-term metal exposure changes gut microbiota of residents surrounding a mining and smelting area. Sci Rep. (2020) 10:44–53. 10.1038/s41598-020-61143-7 32157109 PMC7064573

[44] Jaronczyk K Bui L Soong JM McLaughlin BE Marks GS Brien JF. The source of heme for vascular heme oxygenase - II: *de novo* heme biosynthesis in rat aorta. Can J Physiol Pharmacol. (2004) 82:218–24. 10.1139/y04-015 15181459

[45] Wu GH Zhang JF Zhao QR Zhuang WR Ding JJ Zhang C. Molecularly engineered macrophage-derived exosomes with inflammation tropism and intrinsic heme biosynthesis for atherosclerosis treatment. Angewandte Chemie Int Edition. (2020) 59:4068–74. 10.1002/anie.201913700 31854064

[46] Donzelli S Espey MG Flores-Santana W Switzer CH Yeh GC Huang JM. Generation of nitroxyl by heme protein-mediated peroxidation of hydroxylamine but not N-hydroxy-L-arginine. Free Radical Biol Med. (2008) 45:578–84. 10.1016/j.freeradbiomed.2008.04.036 18503778 PMC2562766

[47] Mendiburo MJ Le Blanc S Espinoza A Pizarro F Arredondo M. Transepithelial heme-iron transport: effect of heme oxygenase overexpression. Eur J Nutr. (2011) 50:363–71. 10.1007/s00394-010-0144-5 21079975

[48] Kumar JS Subramanian VS Kapadia R Kashyap ML Said HM. Mammalian colonocytes possess a carrier-mediated mechanism for uptake of vitamin B3 (niacin): studies utilizing human and mouse colonic preparations. Am J Physiol Gastrointestinal Liver Physiol. (2013) 305:G207–G13. 10.1152/ajpgi.00148.2013 23744738 PMC3742858

[49] Tannous C Booz GW Altara R Muhieddine DH Mericskay M Refaat MM. Nicotinamide adenine dinucleotide: biosynthesis, consumption and therapeutic role in cardiac diseases. Acta Physiologica. (2021) 231:13551. 10.1111/apha.13551 32853469

[50] Stanhewicz AE Kenney WL. Role of folic acid in nitric oxide bioavailability and vascular endothelial function. Nutr Rev. (2017) 75:61–70. 10.1093/nutrit/nuw053 27974600 PMC5155615

[51] Rezk BM Haenen G van der Vijgh WJF Bast A. Tetrahydrofolate and 5-methyltetrahydrofolate are folates with high antioxidant activity. Identification of the antioxidant pharmacophore. FEBS Lett. (2003) 555:601–5. 10.1016/S0014-5793(03)01358-9 14675781

[52] Hill MJ. Intestinal flora and endogenous vitamin synthesis. Eur J Cancer Prevent. (1997) 6:S43–S5. 10.1097/00008469-199703001-00009 9167138

[53] Qi Y Lohman J Bratlie KM Peroutka-Bigus N Bellaire B Wannemuehler M. Vitamin C and B-3 as new biomaterials to alter intestinal stem cells. J Biomed Mater Res Part A. (2019) 107:1886–97. 10.1002/jbm.a.36715 31071241 PMC6626554

[54] Wang Y Jiang M Zhang Z Sun H. Effects of over-load iron on nutrient digestibility, haemato-biochemistry, rumen fermentation and bacterial communities in sheep. J Anim Physiol Anim Nutr. (2020) 104:32–43. 10.1111/jpn.13225 31663652

[55] Halpern D Gruss A. A sensitive bacterial-growth-based test reveals how intestinal Bacteroides meet their porphyrin requirement. BMC Microbiol. (2015) 15:282. 10.1186/s12866-015-0616-0 26715069 PMC4696147

[56] Dschietzig TB Kellner K-H Sasse K Boschann F Kluesener R Ruppert J. Plasma kynurenine predicts severity and complications of heart failure and associates with established biochemical and clinical markers of disease. Kidney Blood Press Res. (2019) 44:765–76. 10.1159/000501483 31387104

[57] Lund A Nordrehaug JE Slettom G Solvang S-EH Pedersen EKR Midttun O. Plasma kynurenines and prognosis in patients with heart failure. PLoS ONE. (2020) 15:0227365. 10.1371/journal.pone.0227365 31923223 PMC6953806

[58] Li QH. Metabolic reprogramming, gut dysbiosis, and nutrition intervention in canine heart disease. Front Vet Sci. (2022) 9:791754. 10.3389/fvets.2022.791754 35242837 PMC8886228

[59] Chen X Li X Xu XD Li LX Liang NN Zhang LL. Ferroptosis and cardiovascular disease: role of free radical-induced lipid peroxidation. Free Radic Res. (2021) 55:405–15. 10.1080/10715762.2021.1876856 33455488

