# Supplementary figures and images for: Combined analysis of plasma metabolome and intestinal microbiome sequencing to explore jiashen prescription and its potential role in changing intestine–heart axis and effect on chronic heart failure
Source: Front Cardiovasc Med. 2023 Mar 10;10:1147438. doi: 10.3389/fcvm.2023.1147438 (PMC10036802; doi:10.3389/fcvm.2023.1147438)

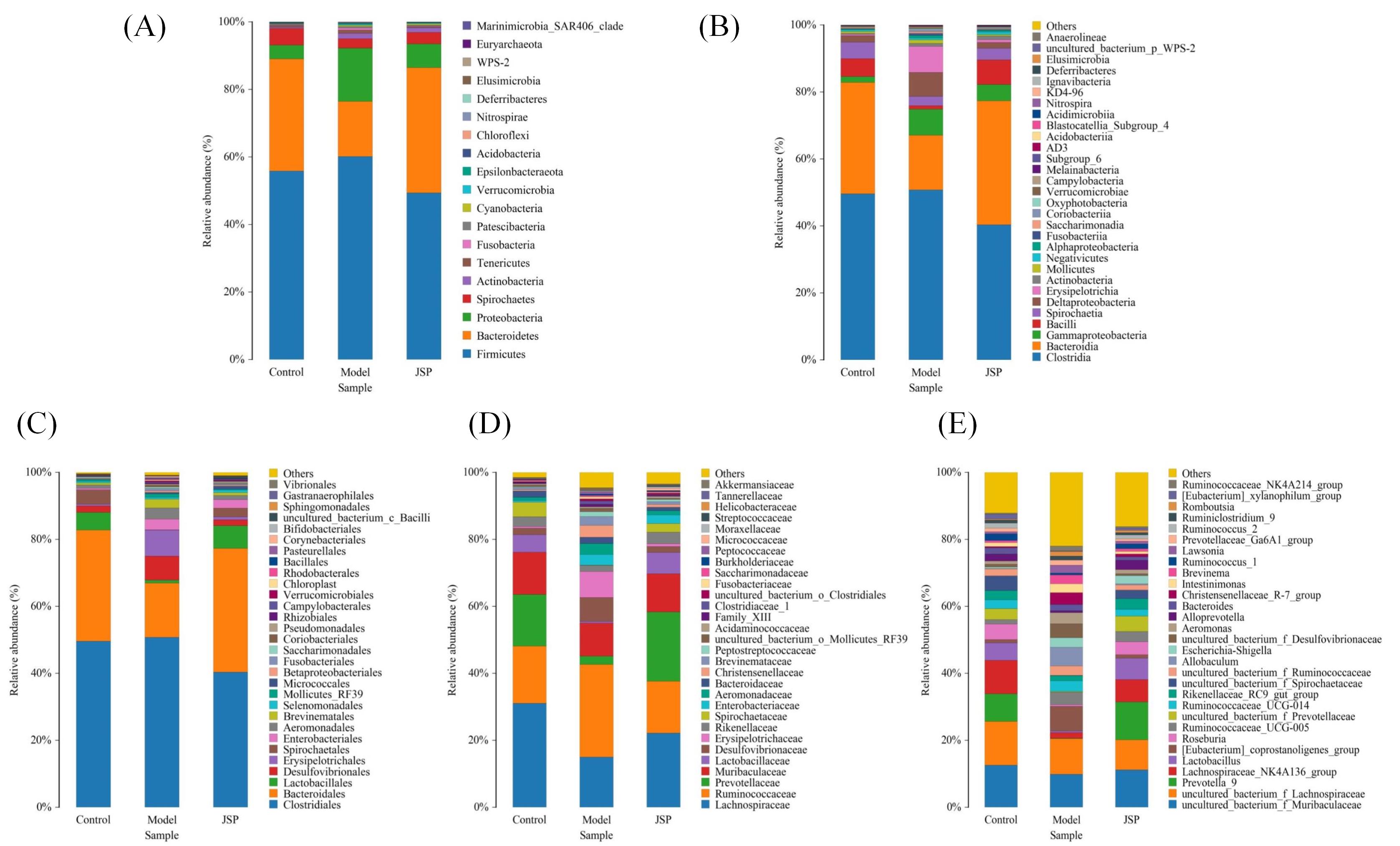

Supplement: Supplementary Figure 1 — The percentage of total bacteria presented at phylum (A), class (B), order (C), family (D), and genus (E) levels. [file Image_1.JPEG]
